# Structure-function investigation of 3-methylaspartate ammonia lyase reveals substrate molecular determinants for the deamination reaction

**DOI:** 10.1371/journal.pone.0233467

**Published:** 2020-05-21

**Authors:** Veronica Saez-Jimenez, Željka Sanader Maršić, Matteo Lambrughi, Jae Ho Shin, Robin van Havere, Elena Papaleo, Lisbeth Olsson, Valeria Mapelli

**Affiliations:** 1 Division of Industrial Biotechnology, Department of Biology and Biological Engineering, Chalmers University of Technology, Gothenburg, Sweden; 2 Computational Biology Laboratory, Danish Cancer Society Research Center, Copenhagen, Denmark; Bogazici University, TURKEY

## Abstract

The enzymatic reactions leading to the deamination of β-lysine, lysine, or 2-aminoadipic acid are of great interest for the metabolic conversion of lysine to adipic acid. Enzymes able to carry out these reactions are not known, however ammonia lyases (EC 4.3.1.-) perform deamination on a wide range of substrates. We have studied 3-methylaspartate ammonia lyase (MAL, EC 4.3.1.2) as a potential candidate for protein engineering to enable deamination towards β-lysine, that we have shown to be a competitive inhibitor of MAL. We have characterized MAL activity, binding and inhibition properties on six different compounds that would allow to define the molecular determinants necessary for MAL to deaminate our substrate of interest. Docking calculations showed that β-lysine as well as the other compounds investigated could fit spatially into MAL catalytic pocket, although they probably are weak or very transient binders and we identified molecular determinants involved in the binding of the substrate. The hydrophobic interactions formed by the methyl group of 3-methylaspartic acid, together with the presence of the amino group on carbon 2, play an essential role in the appropriate binding of the substrate. The results showed that β-lysine is able to fit and bind in MAL catalytic pocket and can be potentially converted from inhibitor to substrate of MAL upon enzyme engineering. The characterization of the binding and inhibition properties of the substrates tested here provide the foundation for future and more extensive studies on engineering MAL that could lead to a MAL variant able to catalyse this challenging deamination reaction.

## Introduction

In order to move from an oil-based economy to a bio-based economy, reliable processes must be established for the production of a broad range of chemicals important in modern societies. Adipic acid is one of such chemicals and is used mainly as a building block for nylon polymers [1,2]. The global demand for adipic acid is high; around three million tons per year are produced by chemical synthesis using petrochemical resources [3]. This chemical process results in the emission of greenhouse gases and environmental pollution. Therefore, replacing this chemical process with a bio-based one using renewable feedstocks would constitute a sustainable alternative [4,5].

Extensive research efforts have been made to design cell factories able to produce adipic acid from various resources, mainly carbohydrates, but the so far obtained titers, productivities and yields must be improved to be industrially relevant [3,6,7]. In the present work, we focus on the study of metabolic pathways that could be used to produce adipic acid from lysine. Lysine has been ranked among the top 30 building blocks, according to factors such as suitability for further conversion and the possibility of production from biomass [8–10]. Lysine is currently produced on large scale using a genetically engineered *Corynebacterium glutamicum* [11], paving the way for the bio-based production of chemicals derived from lysine.

**Fig 1:**
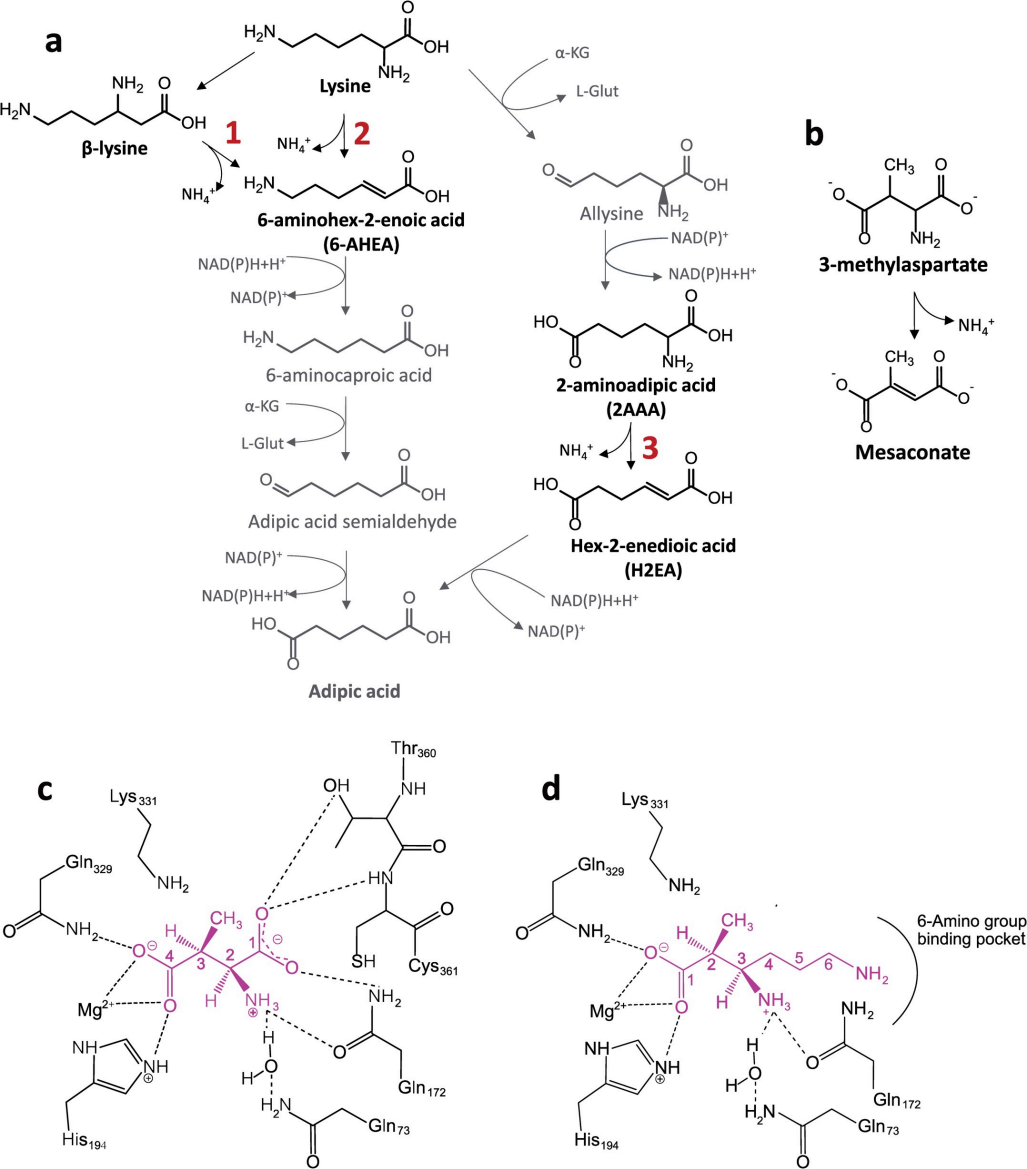
Deamination reactions in metabolic pathways for adipic acid biosynthesis. (a), Metabolic pathway(s) considered for the production of adipic acid from lysine. The three deamination reactions are numbered in red. (b), Reaction catalysed by 3-methylaspartate ammonia lyase (MAL). (c), Schematic representation of 3-methylaspartate (magenta, with carbon labelling indicated) bound in the CaMAL catalytic pocket (black). Adapted from [14]. (d), Schematic representation of our hypothesis on how β-lysine (magenta, with carbon labelling indicated) would bind in CaMAL catalytic pocket (black).

The production of adipic acid from lysine involves at least four enzymatic reactions (Fig 1A) [12]. Two pathways, both involving the same kind of reactions but in different sequence, have been proposed (Fig 1A, pathways including reactions 2 and 3) [13]. Here, we propose an additional variant of the metabolic pathway, wherein lysine is converted into β-lysine via lysine 2,3-aminomutase (EC 5.4.3.2), and β-lysine is then deaminated to 6-AHEA (Fig 1A, reaction 3). The main challenge that must be overcome in these pathways is the lack of known efficient enzymes for some of the metabolic reactions. This is the case for the deamination reactions leading to the deamination of β-lysine or lysine to 6-aminohex-2-enoic acid (6-AHEA) (Fig 1A, reaction 1 and 2) or to the deamination of 2-aminoadipic acid (2AAA) to hex-2-enedioic acid (H2EA) (Fig 1A, reaction 3) [13]. All these deamination reactions are critical in the above mentioned metabolic pathways, and it is therefore essential to identify or engineer enzymes able to carry out these reactions for the implementation of these pathways.

In order to solve this issue, we focused our attention on a number of enzymes able to deaminate amino acids. In particular, we considered aspartate-, histidine-, and 3-methylaspartate ammonia lyase (MAL). However, the present work is focused on MAL (EC 4.3.1.2) which catalyses the conversion of 3-methylaspartate to mesaconate (Fig 1B) through an α,β-elimination mechanism. MALs are well-known enzymes that were first described in some facultative anaerobic bacteria [15–17] as part of the catabolic pathway for the transformation of L-glutamic acid to acetyl-coenzyme A. MAL is also part of the anabolic methylaspartate cycle in haloarchaea [18]. The best-characterized MALs are those produced by *Citrobacter amalonaticus* (CaMAL) and *Clostridium tetanomorphum* (CtMAL) [19–21]. Their crystal structures reveal that MALs are homodimers (45 kDa) belonging to the enolase superfamily [22,23]. The catalytic mechanism involves the abstraction of a proton from the carbon atom in position 3 (C3 position) by a base catalyst (K331 residue, Fig 1C) which leads to the formation of an enolate intermediate, which is stabilized by interactions with the Mg^2+^ ion, Q329 and H194. The collapse of this intermediate is followed by elimination of ammonia from the C2 position [23]. We propose here the deamination of β-lysine, that shares a common structural feature with 3-methylaspartate, that is a carboxyl group followed by a non-polar group (i.e., -CH_2_- or with a hydrogen substituted by a -CH_3_ group) and then by the carbon atom with the amino group attached. In particular, we hypothesize that β-lysine could bind MAL in a similar way as 3-methylaspartate does (Fig 1D), with the carboxylic group interacting with the Mg^2+^ ion, Q329, and H194. In this way, the amino group on carbon 6 would be facing T360 and C361, as the α-carboxylic group of 3-methylaspartate (carboxylic group at C1) does. Then the proton from carbon 2 of β-lysine (corresponding to carbon 3 in 3-methylaspartate) could be abstracted by K331, the enolate intermediate would be formed and the amino group on carbon 3 would be eliminated.

**Fig 2:**
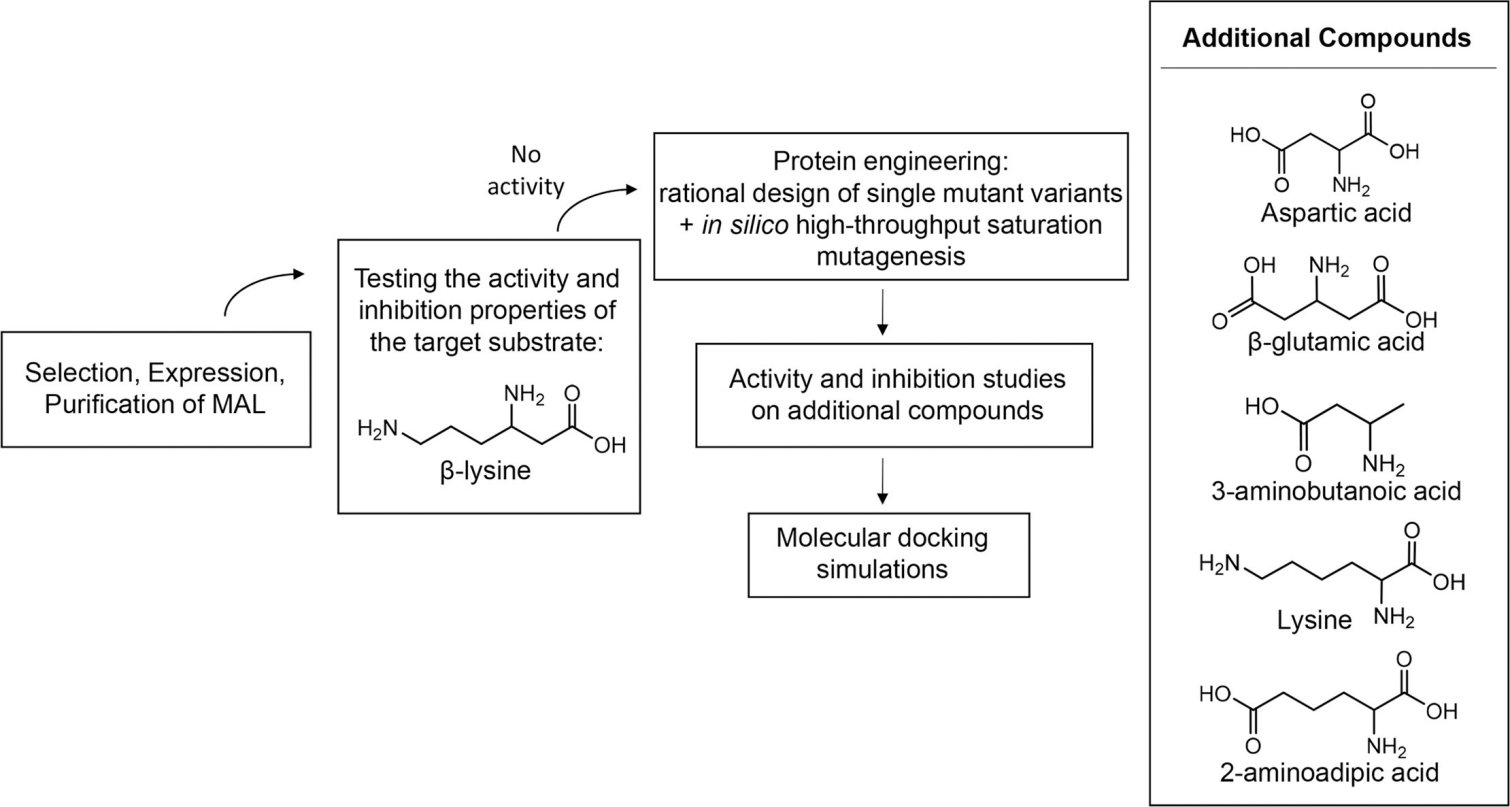
Strategies and workflow employed in the present study. The activity and inhibition of three different MALs on the target substrate β-lysine was assayed. A protein engineering strategy combining rational design with an *in silico* high-throughput saturation mutagenesis method was then used to design and test five single-mutant variants of CaMAL. Finally, molecular docking, and activity and inhibition assays were carried out in additional substrates in order to characterize the molecular requirements for the substrate binding in MAL.

Here we address two main challenges: i) to assess the potential of MAL to catalyze the deamination of β-lysine and ii) to identify the molecular determinants of the MAL substrate crucial for the binding to the catalytic pocket and for the deamination mechanism, hence iii) to evaluate the possibilities of engineering MAL for the deamination of our target substrate. The strategy adopted to address these challenges is illustrated in Fig 2. Three MALs from three different species were produced recombinantly, and their activity towards the target substrate was investigated *in vitro*. In particular, we produced and tested CaMAL, CtMAL, and ChMAL (MAL from *Carboxydothermus hydrogenoforomans*). A protein engineering strategy combining a rational design with *in silico* high-throughput saturation mutagenesis was used to design mutant variants that could be potentially able to deaminate β-lysine. Finally, a combination of molecular docking and *in vitro* assays was subsequently used to investigate and characterize the binding and inhibition properties of five additional compounds. The selected compounds are in fact characterized by specific chemical features that would allow to understand the molecular determinants that are involved in the effective binding of the substrate in MAL.

## Results

### Three different MALs were selected, expressed and purified

The well-characterized MAL enzymes CaMAL, CtMAL and ChMAL were selected based on the extensive information available on their catalytic mechanism, their biochemical properties and the availability of their crystal structures [22,23]. Moreover, CtMAL has previously been engineered to broaden its substrate specificity to synthesize different aspartic acid derivatives [24]. CaMAL and CtMAL share a degree of sequence identity of 58%. ChMAL was also studied in the present work since it has been biochemically characterized, and has a degree of sequence identity of 53% and 52% with CaMAL and CtMAL, respectively. Although the residues involved in the catalysis and binding of the substrate of the three selected MALs are conserved, the relatively low sequence identity might increase the chances of finding different substrate specificities towards our target substrate.

Recombinant CaMAL, CtMAL and ChMAL were produced in *Escherichia coli* and showed activity towards the natural substrate 3-methylaspartate (Table 1). The kinetic constants obtained were comparable to those reported previously [21,25,26]. This confirmed that CaMAL, CtMAL and ChMAL were correctly folded and that the histidine tag they harbour at the C-terminal tail did not interfere with their catalytic activity.

**Table 1:** Steady-state kinetic constants for the activity of MAL enzymes on 3-methylaspartic acid and aspartic acid.

	Substrate	*k*_cat_ (s^-1^)	*K*_m_ (mM)	*k*_cat_ (s^-1^) / *K*_m_ (mM)
CaMAL	3-Methylaspartic acid	17.6 ± 0.3	1.00 ± 0.06	17.6
	Aspartic acid	4.4 ± 0.4	141 ± 14	0.031
ChMAL	3-Methylaspartic acid	66 ± 2	1.38 ± 0.17	47.8
	Aspartic acid	1.1 ± 0.6	171 ± 107	0.006
CtMAL	3-Methylaspartic acid	7.6 ± 0.4	3.99 ± 0.61	1.9
	Aspartic acid	1.6 ± 0.1	67 ± 6	0.023

Values and standard errors for apparent affinity constant (Michaelis constant, *K*_m_) and maximal enzyme turnover (catalytic constant, *k*_cat_), and values for enzyme efficiency values (*k*_cat_ /*K*_m_). Reactions at 30°C in 0.25 M Tris pH 9, 20 mM MgCl_2_ and 1 mM KCl. Means and 95% confidence limits.

### β-lysine is a competitive inhibitor of MAL

To investigate the activity of the recombinant MALs on the target substrate β-lysine, a spectrophotometric assay was developed based on the comparison of the absorption spectra of the target substrate and the corresponding product of the deamination reaction. The product 6-AHEA showed higher absorbance in the UV region (220–260 nm) than β-lysine (S1 Fig). Specifically, the absorbance of 6-AHEA was 175 times higher than that of β-lysine at 230 nm. The extinction coefficient of 6-AHEA at 230 nm was found to be 5240 M^-1^cm^-1^. In view of these findings, the deamination of β-lysine, was assayed by monitoring the possible formation of 6-AHEA at 230 nm. No increase in the absorbance at 230 nm was observed when monitoring the reaction. Therefore, we concluded that MAL could not catalyse the deamination of β-lysine under the conditions tested (S2 Fig). However, the amount of enzyme used was limited to around 20 μg/ml, as the absorption of the MAL at 230 nm interfered with the assay.

**Fig 3:**
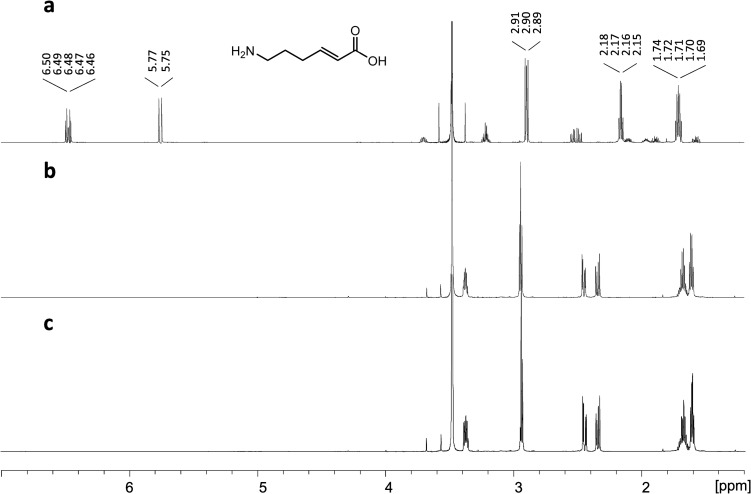
MAL activity on β-lysine monitored using ^1^H NMR. (a), Spectrum corresponding to 60 mM 6-AHEA in reaction buffer. (b), Spectrum corresponding to β-lysine incubated with CaMAL at 30°C for 4 d in reaction buffer. (c), Spectrum corresponding to the control reaction in which β-lysine was incubated in reaction buffer for 4 d at 30°C.

To detect minute amounts of the deamination products, other methods were tested that allowed the use of higher enzyme concentrations or higher concentrations of substrate. Therefore, the deamination of β-lysine to 6-AHEA was monitored using NMR. The ^1^H NMR spectrum of 6-AHEA was obtained (Fig 3A) and compared with the spectrum of β-lysine incubated in the presence of CaMAL (Fig 3B), but it did not show any new signal corresponding to 6-AHEA. Moreover, it was almost identical to that of the control reaction without enzyme (Fig 3C), which indicated that no enzymatic reaction took place.

**Fig 4:**
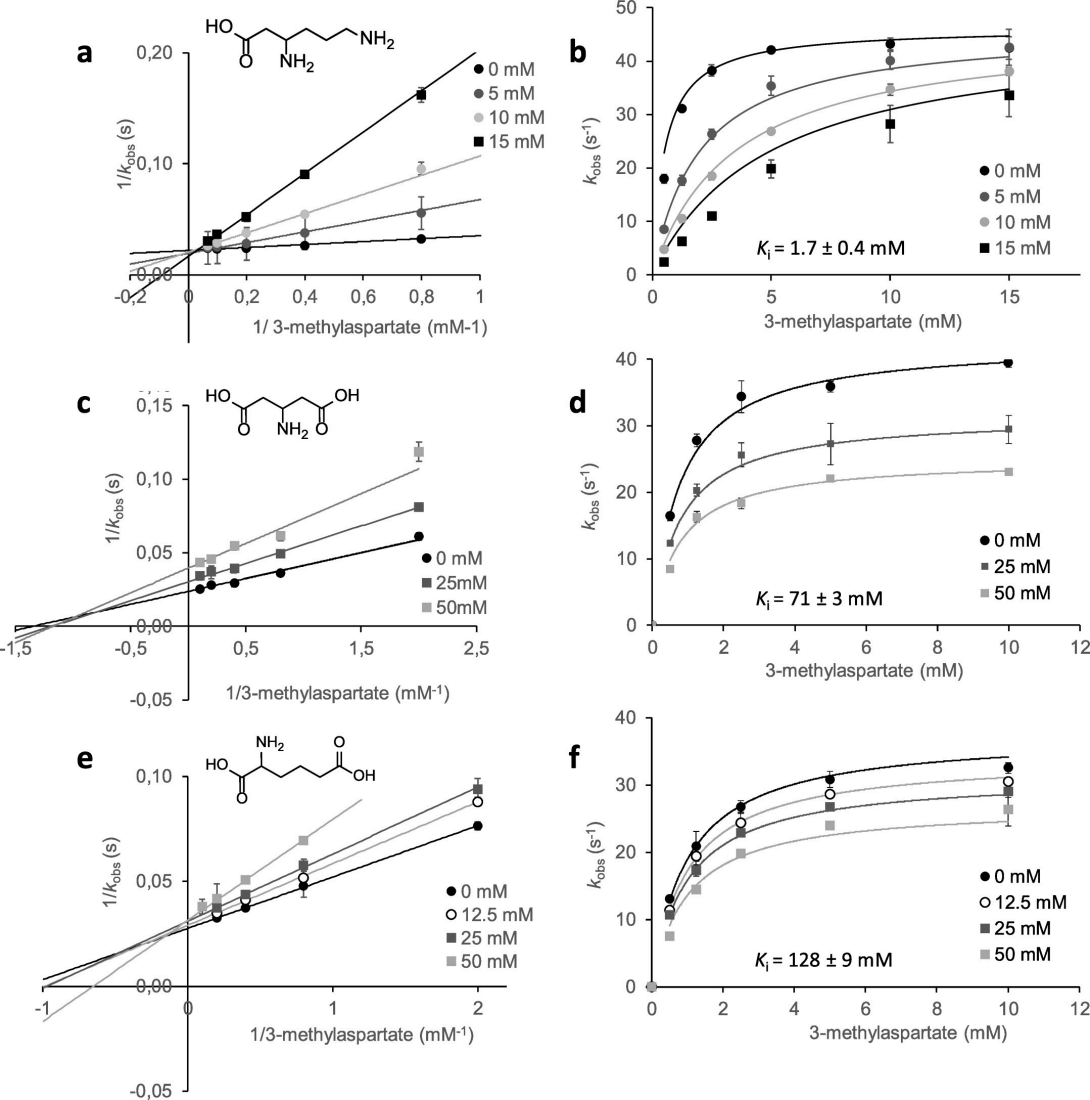
Inhibition assays. Lineweaver-Burk plots of 3-methylaspartate deamination by CaMAL in the presence of different concentrations of β-lysine (a), β-glutamic acid (c) and 2AAA (e). Plots of 3-methylaspartate deamination by CaMAL in the presence of different concentrations of β-lysine (b), β-glutamic acid (d) and 2AAA (f) and fitting to a non-linear regression function (competitive inhibition function following the equation y = *k*_cat_ x / *K*_m_ (1 + *I*_c_/ *K*_i_) + x or noncompetitive inhibition function following the equation y = *k*_cat_ x / (1 + *I*_c_/ *K*_i_) (*K*_m_ + x)). β-lysine data fitted to the competitive inhibition function (Global R^2^ = 0.998), β-glutamic acid data fitted to the non-competitive inhibition function (Global R^2^ = 0.995), and 2-aminoadipic acid data fitted to the non-competitive inhibition function (Global R^2^ = 0.997). Inhibition constants (*K*_i_) of CaMAL obtained for the three inhibitors are shown. Data are the means of three replicates with 95% confidence limits.

Since no activity was detected, the possible inhibition of MAL by β-lysine was investigated using CaMAL as the model enzyme (Fig 4A and 4B). The double reciprocal plots of the initial velocity versus 3-methylaspartate concentration with increasing β-lysine concentration showed a typical competitive inhibition pattern (Fig 4A), that indicates that β-lysine is able to bind in the catalytic pocket of CaMAL, competing with 3-methylaspartate. The fitting of the data to a non-linear regression model for competitive inhibition function also supported the that β-lysine is a competitive inhibitor (Fig 4B). Our results showed that β-lysine was an inhibitor of CaMAL featured by a *K*_i_ value of 1.7 ± 0.4 mM, that is a value comparable to *K*_m_ for 3-methylaspartate (1.0 ± 0.1 mM, Table 1).

### The design of single-mutant variants did not lead to activity on β-lysine

Following a rational strategy and using the information available in the literature [21,24,27,28], five single mutant variants were designed that could modify the accommodation of β-lysine in the catalytic pocket and potentially enable activity on β-lysine.

The residues T360 and C361 interact with the carboxylate group at C1 of 3-methylaspartate in the CaMAL crystal structure (Fig 1C) through the oxygen atom (Oγ) and the backbone (NH group), respectively. Since β-lysine has a two carbon longer side chain compared to 3-methylaspartate and has an amino terminal group instead of the carboxylate at C1 in 3-methylasparate, we decided to mutate T360 and C361 by smaller residues (serine and alanine) with the intention of creating more space in the catalytic pocket to accommodate the two extra carbons and the terminal amino group of β-lysine side chain. Interestingly, the substitution of C361 by alanine in CtMAL was previously shown not to affect the kinetic constants to any great extent [27].

The residue L384 is on the surface of the protein lining the catalytic pocket and has been suggested to contribute to a hydrophobic pocket that interacts with the methyl group of 3-methylaspartate [23]. In addition, it has already been found that the CtMAL L384A variant has broader substrate specificity than the native MAL [24] showing the importance of this residue in the catalytic pocket. For these reasons, the single-mutant variants T360A, T360S, C361A, C361S and L384A were selected as potential candidates to be studied experimentally.

Subsequently, to evaluate the local effects of the selected amino acidic substitutions on the structure of CaMAL and exclude the ones that would affect its stability, we used an *in silico* high-throughput saturation mutagenesis scan based on the *FoldX* energy function [29, 30]. It has been shown that FoldX is effective in capturing destabilizing mutations in proteins [31]. We employed this method to assess the impact of mutations on the thermodynamic stability of CaMAL calculated as ΔΔG value (S1 Table). We observed that most of the CaMAL mutant variants were predicted with only moderate destabilizing effects on CaMAL, therefore the mutant variants were expressed, purified, and the activity tested experimentally.

All the single-mutant variants showed activity towards 3-methylaspartate (S2 Table). The CaMAL T360S and C361S variants had kinetic constants comparable to those of native CaMAL (Table 1). CaMAL C361A showed a 9.7-fold decrease in *k*_cat_ and a similar value of *K*_m_, which led to a slight decrease in catalytic efficiency (a 6-fold decrease). The mutations T360A and L384A, on the other hand, led to reductions in the *k*_cat_ values (by 217-fold and 1760-fold, respectively). The activity of CaMAL single mutants towards β-lysine was monitored following 6-AHEA formation at 230 nm (S3 Fig) but, unfortunately, no activity was detected with the methodology used.

### The methyl group in 3-methylaspartate is an important factor for the affinity of the substrate

Since no deamination of β-lysine was detected with the native or engineered MALs, five additional compounds showing or lacking specific chemical features compared with 3-methylaspartate and β-lysine (Fig 2, right panel) were tested to elucidate which molecular determinants a MAL substrate must have, and to broaden our knowledge on the substrate specificity of the enzyme.

One of the compounds tested was aspartic acid, which lacks the methyl group present in 3-methylaspartic acid (Fig 2, right panel). Although CaMAL has previously been shown not to catalyse the deamination of aspartic acid [26,32], we found that both CaMAL and ChMAL deaminated aspartic acid (Table 1). The apparent affinity constants (*K*_m_) exhibited a remarkable increase, of 42- to 141-fold, compared with the respective *K*_m_ values obtained for 3-methylaspartic acid. In contrast, the maximal enzyme turnover (*k*_cat_) values were impaired to a lesser extent, showing a decrease of 4-, 41-, and 7-fold for CaMAL, CtMAL and ChMAL, respectively. These results provide evidence that the affinity for the substrate is highly impaired when aspartic acid is used as a substrate, and suggest that the methyl group of 3-methylaspartate is a determinant factor for the effective binding of the substrate to MAL, and subsequent efficient catalysis.

To understand how critical the presence of the α-carboxylate group is in the native substrate and in aspartic acid, 3-aminobutanoic acid was tested as substrate, as it lacks the α-carboxylate group (Fig 2, right panel). However, no significant MAL activity was detected on 3-aminobutanoic acid (S3 Table).

Furthermore, β-glutamic acid was tested as MAL substrate. β-glutamic acid is a dicarboxylic acid with one extra carbon in the backbone compared to aspartic acid (Fig 2, right panel). The analysis of the deamination ability of MAL on β-glutamic acid would help to understand whether a 5-carbon dicarboxylic substrate can be deaminated by MAL. In order to follow the enzymatic reaction, a spectrophotometric assay was developed based on the difference in absorption at 210 nm between β-glutamic acid and its deamination product glutaconate (S1 Fig). In fact, in the HPLC method developed both β-glutamic acid and glutaconic acid had the same retention time (3.85 min, S4A Fig), however the high sensitivity of the method for detecting glutaconic acid (100 μM glutaconic acid could be detected in the presence of 60 mM β-glutamic acid) compared with the poor absorbance of β-glutamic acid, made it a suitable method for monitoring this deamination reaction. When MALs were incubated with β-glutamic acid for 14 days, no significant increase was observed in the peak at 3.85 min compared with the control reactions (S4 Fig), indicating a lack of activity.

Besides, the inhibition of β-glutamic acid on MAL was studied using CaMAL as the model enzyme (Fig 4C and 4D). The double reciprocal (Lineweaver-Burk) plot (Fig 4C) revealed a profile suggesting the occurrence of non-competitive inhibition by β-glutamic acid. The same results were obtained when the data were fitted by non-linear regression to the non-competitive function (Fig 4D). Our results showed that β-glutamic acid is a weak inhibitor of CaMAL, with *K*_i_ = 71 ± 3 mM.

Finally, although lysine and 2AAA are improbable MAL substrates due to mechanistic issues, and a complex redesign of the catalytic machinery would be needed for substrate conversion, we considered these molecules as MAL inhibitors. In fact, the study of the inhibition by lysine having the amino group in carbon 2 (equivalent to the position of the methyl group in 3-methylaspartate) differing from β-lysine, that has the amino group bound to carbon 3 (equivalent to the position of the amino group in 3-methylaspartate) could give useful information about the role this specific amino group in the binding. The presence of different concentrations of lysine (up to 100 mM) did not affect CaMAL activity (S5 Fig), while 2AAA was a weak non-competitive inhibitor (Fig 4E and 4F), with a *K*_i_ value of 128 ± 9 mM.

### Docking analysis: All the compounds tested fit spatially in the MAL catalytic pocket

**Fig 5:**
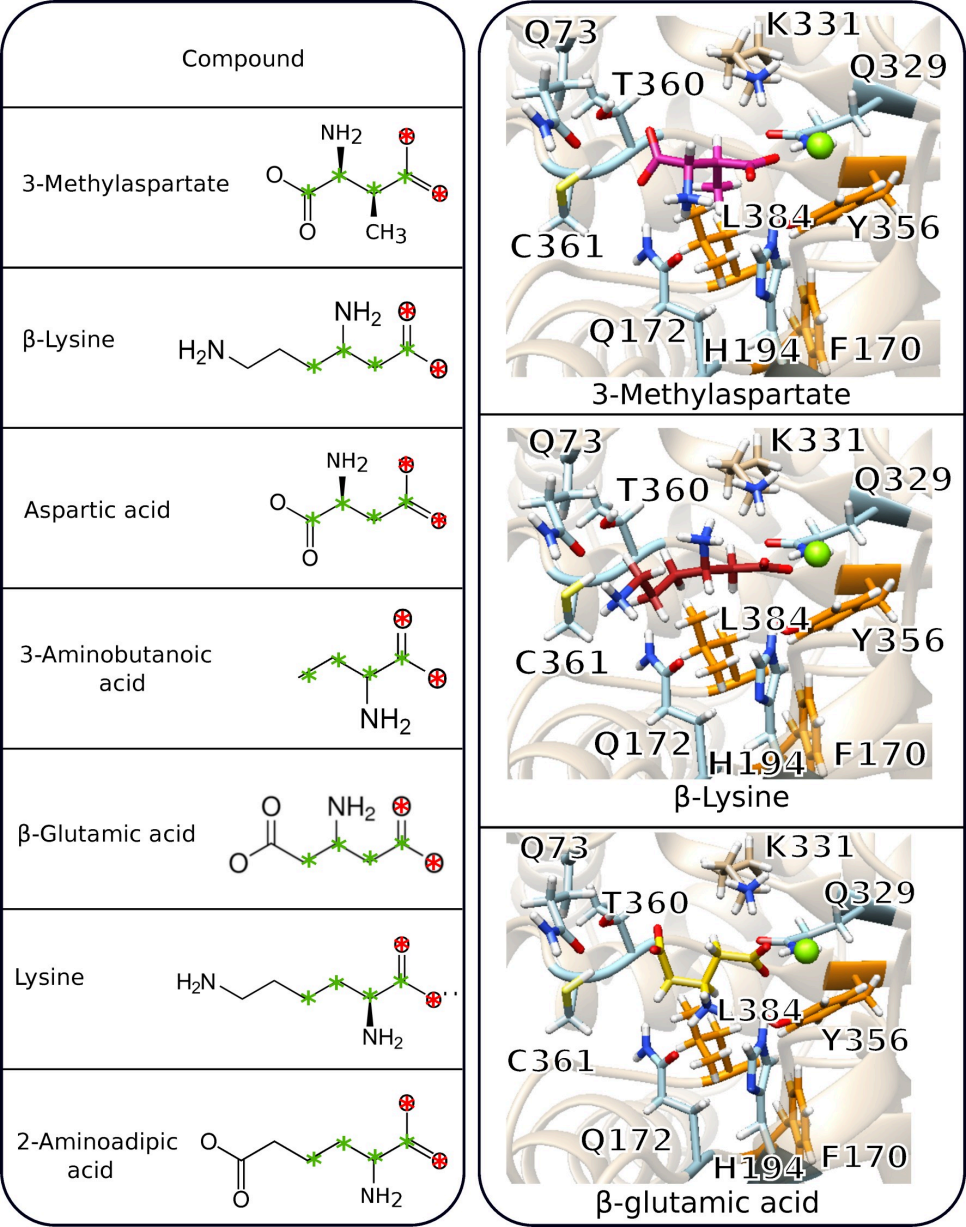
Results of docking analysis. The structures of the natural substrate (3-methylaspartate) and the investigated substrates are shown in the left panel, together with the description of their structural differences compared with the natural substrate. The carbon atoms, indicated in green, and the oxygen atoms, in red, constitute a common scaffold in all the substrates, and these atoms were used for the RMSD calculations. The right panel shows the catalytic pocket of CaMAL (shown as the pale brown cartoon) with the natural substrate (upper panel, PDB entry 1KKR, shown in magenta), one example of the complexes with lysine (middle panel, shown in light pink) and β-lysine (lower panel, shown in red) obtained by docking calculations. difference in the orientation of the ligands with respect to the hydrophobic residues in the catalytic pocket of CaMAL (residues L384, F170, Y356 shown as orange sticks) and the residue Q172 (shown as blue sticks), which takes part in the hydrogen bond network. The interactions predicted for the other ligands in the catalytic pocket of CaMAL are shown in S7 Fig.

To obtain possible models of the three-dimensional structure of the complexes between the six experimentally characterized ligands and MAL, we used molecular docking. The best poses were selected according to two criteria: i) the distances between the ligand and key residues in the substrate binding pocket, and ii) the root mean square deviation (RMSD) between the ligands and the natural substrate (see left panel in Fig 5). As a result of applying the first criterion, we found that 81% of lysine, 80% of β-lysine, 78% of β-glutamic acid, 55% of aspartic acid, 68% of 3-aminobutanoic acid, and 90% of 2AAA poses were inside the binding pocket. These results indicate that all six ligands can fit spatially inside the binding pocket of CaMAL.

The calculated RMSDs between the ligands and the position of the natural substrate in the reference X-ray structure were used as an additional criterion to select the poses (see Methods). The RMSD values ranged from 1.5–4 Å (S6 Fig). The pose with the lowest RMSD value relative to the positioning of the natural substrate for each ligand was selected. Notably, the selected poses featured better estimated binding affinities than the first poses proposed according to the docking ranking algorithm (S4 Table).

As the catalytic mechanism of MAL includes proton transfer from the carbon atom in position 3 of the 3-methylaspartic acid [21], the correct positioning of the ligand inside the binding pocket is important for the reaction. We thus studied the intermolecular interactions between the enzyme and the ligands in the selected docking poses using the Arpeggio software. To appreciate the differences in binding between the natural substrate and the various ligands, we used the binding mode of the natural substrate as a reference. The binding pocket of CaMAL includes the residues Q73, Q172, H194, Q329, K331, G359, T360, C361, and M389 (featured residues in the right panel of Fig 5 and S7 Fig). The most relevant interactions that have been reported are hydrogen bonds, which require the residues Q172, H194, Q329, C361 and T360, as well as, via a water molecule, Q73 [23]. Functional screening of mutants of MAL have shown that the residues mentioned above are important for the correct positioning and activation of the substrate, and that they are involved in the specificity of CaMAL [27]. The Arpeggio analysis of the interactions formed by these residues and the 3-methylaspartate confirmed the network of hydrogen bonds listed above (S8 Fig), except for H194 and Q73. These two interactions were classified by Arpeggio as ionic interaction for H194 and proximal interaction for Q73 as the tool does not account for the water molecule. Arpeggio identified also additional interactions involving the residues D238, Y240, E273, D307 and C361 and the Mg^2+^ ion. Also, a hydrophobic interaction was found between the methyl group of the substrate and L384 of CaMAL (highlighted in orange in Fig 5).

To provide a model at the molecular level of how the different ligands act, or do not act, as substrates/inhibitors of CaMAL activity, the substrate binding pocket was investigated in more detail. We thus compared the intermolecular interactions observed for the complexes of CaMAL with the six ligands investigated to the reference structure of the complex of CaMAL with the natural substrate (right panel in Fig 5 and S7 and S8 Figs). Some of the ligands, such as aspartic acid, β-lysine and β-glutamic acid, have a structure with a carboxyl group followed by a non-polar group (i.e., -CH_2_-), or with a hydrogen substituted by a -CH_3_ group, as in 3-methylaspartate, followed by the carbon atom with the amino group attached. Our analysis suggests that these ligands could bind in a similar way as the natural substrate 3-methylaspartate, establishing important and necessary interactions since they present a non-polar group (-H or -CH_3_) correctly oriented towards the hydrophobic pocket, and the amino group is accessible for hydrogen bonding. The complex of CaMAL with the natural substrate did in fact show the presence of a hydrophobic interaction with the -CH_3_ group; while in the complex with β-lysine the hydrophobic interaction included the -CH_2_- group (S8 Fig). In contrast, lysine has the amino group on the carbon next to the carboxyl one, and, as expected, hydrophobic interactions were not observed in the analysis. Similarly, β-lysine can provide the same hydrogen bonds formed by the -NH_2_ group of the natural substrate. Arpeggio analysis showed that the amino group of the aspartic acid and β-glutamic acid are involved in similar contacts with CaMAL as the natural substrate and β-lysine (S8 Fig), further supporting the proposed model. Hydrogen bonds are also compatible with the complex between CaMAL and lysine, but they involve different residues.

## Discussion

The main problem to overcome before the proposed metabolic pathways can be introduced in a suitable production host to produce adipic acid from lysine is the lack of an enzyme able to carry out the deamination reaction [7,12,13]. In the present work, we suggest a new variant of the lysine metabolic pathway for the production of adipic acid that includes the synthesis of β-lysine from lysine (Fig 1A) via 2,3-aminomutase (EC 5.4.3.2) [33]. β-lysine could then be deaminated to 6-AHEA via an ammonia lyase. Since MAL is able to deaminate 3-mehtylaspartate, a substrate with a similar chemical structure as β-lysine, here we have investigated the potential of MAL or possible mutant variants to deaminate β-lysine.

The analysis of the *in silico* and *in vitro* results improved our understanding of the different effects of the ligands tested on enzyme activity and binding. Interestingly, the *in silico* studies indicated that the catalytic pocket is sufficiently large to accommodate the tested substrates, which are mostly more voluminous than 3-methylaspartate. For instance, ββ-lysine has one extra carbon compared with MAL natural substrate. However, the analysis of the binding affinities of the seven poses studied showed lower binding affinity than the natural substrate for all the studied ligands. This suggests that although the ligands can fit into the catalytic pocket, they may be weak or very transient binders. It is important to note that the docking results should be interpreted with caution. In fact, although static docking is a fast and convenient computational approach, it cannot account for all the aspects that are needed to fully understand the binding of the substrates and the activity of the enzyme on different substrates. For example, due to the fact that we define a grid around the catalytic site to carry out the docking, the possibility to predict that the CaMAL non-competitive inhibitors 2-aminoadipic acid or β-glutamic acid can bind to a region different from the catalytic site is not possible with this approach. Also, docking does not provide a dynamic view of the system, or information on the encounter complex, where charged residues could favour or disfavour the ingress of the substrate into the binding pocket. Moreover, we cannot simulate association and dissociation between the enzyme and the ligands and account for the reactivity. Such studies will require a long-term approach and exhaustive calculations with a plethora of approaches [34–37], for which this study has laid the foundations.

On the other hand, the *in vitro* activity assays showed aspartic acid to be the only non-natural substrate of MAL among the substrates analysed; even though β-lysine and β-glutamic acid also have a structure that is compatible with MAL catalytic mechanism. CtMAL activity towards aspartic acid has been described previously [32] and here we show that also CaMAL and ChMAL are active towards aspartic acid, although the kinetic constants were lower than those of 3-methylaspartic acid as described for CtMAL (Table 1). The *K*_m_ values were the most affected catalytic constant, which indicates that the affinity of MAL for aspartic acid is much lower than the affinity for 3-methylaspartic acid. Since the only difference between aspartic acid and 3-methylaspartic acid is the presence of the methyl group in the latter, this group must play a crucial role in the binding and correct orientation of the substrate in the binding pocket. The residues Y356, L384 and F170 create a hydrophobic pocket that accommodates the 3-methyl group of the substrate, forming stabilizing interactions [23]. Indeed, the mutants of Y356 and L384 (Y356A and L384A) show a significant increase on the *K*_m_ of MAL [24,27], showing that these residues play an important role in the binding affinity of the substrate. In agreement with this, it has been suggested previously that the absence of the methyl group in aspartic acid could lead to a change in the conformation of the substrate that hinders the abstraction of the acidic proton or the rupture of the C-N bond [32], therefore slowing down the reaction. Interestingly, although β-lysine lacks the methyl group, hydrophobic interactions were predicted to be formed through the CH_2_ group alpha to the carboxylic group.

Moreover, although β-lysine did not function as MAL substrate, this compound was predicted to be geometrically able to fit and bind into the catalytic pocket, competing with 3-methylaspartate, as was demonstrated by the inhibition assays. The comparison of the inhibition effect done by β-lysine and lysine gave useful information on the role of the amino group on the binding. Interestingly, lysine did not inhibit CaMAL. Since the only difference between β-lysine and lysine is the position of the amino group located on carbon 3 in β-lysine (corresponding to position of the amino group in 3-methylaspartate) and on carbon 2 in lysine (corresponding to position of the methyl group in 3-methylaspartate), the fact that β-lysine has the amino group on the carbon 3 seems to be determinant for the binding of the substrate and not only for the catalytic mechanism. The residues Q172, and Q73, through a water molecule, have been proposed to form hydrogen bonds with the amino group of the natural substrate (blue residue in Fig 5) [23]. Mutations of Q172 have considerable effects on *K*_m_ (Q172A) and may almost completely abolish the catalytic activity (Q172N). Protein engineering studies on MAL based on functional screening of mutants for the amination or deamination of non-natural substrates, have shown that the residues discussed above are important for the correct positioning and activation of the substrate, and are involved in the specificity of MAL [27].

Although the attempts to find a MAL variant that catalyzes the deamination of β-lysine were not successful, the fact that the catalytic pocket is ample enough to accommodate the substrate and the fact that the inhibition assays suggest that it is binding into the catalytic pocket makes MAL a promising enzyme to be engineered. Although the chemical structure of β-lysine is suitable for binding MAL catalytic pocket, the presence of the terminal amino group of β-lysine (replacing the α-carboxylic group of 3-methylaspartate) may require the substitution of two or more residues in the active site to form a stronger network of interactions enabling a better accommodation and binding of the substrate and allowing the catalytic reaction to happen. The mutations should be designed in the region interacting with, or in the vicinity of the α-carboxylate group of 3-methylaspartate, where the terminal group of β-lysine would fit. Since a rational design of the mutation or mutations needed to obtain a correct accommodation of β-lysine and activity is difficult to achieve, other more powerful methods could be applied in order to find a MAL variant able to catalyse the deamination of β-lysine. For example, machine learning methods have been proven to be successful in broadening the substrate specificity of different enzymes [38,39]. Machine learning algorithms are able to predict the structure, folding, binding, or catalytic activity of a target protein using accumulated information about mutants and their properties. The data obtained from protein engineering experiments such as directed evolution or site-specific mutagenesis could serve as training set for these algorithms to facilitate the prediction of new and improved MAL variants. Alternatively, the use of *de novo* protein design could be an interesting option considering the absence of any MAL activity on β-lysine. *De novo* protein design was recently successfully applied to broaden the substrate specificity of aspartase [40]. This method is based on the introduction of amino acid residues essential for catalysis into the existing scaffolds through the exploration of a huge sequence and conformation space. This method could be combined with directed evolution methods for fine-tuning of the constructs obtained *in silico*.

In conclusion, we believe that the characterization of the binding and inhibition properties of the substrates shown here provide the foundation for future and more extensive studies on engineering MAL that could lead to a MAL variant able to catalyse deamination of non-natural substrates for the biosynthesis of molecules of interest.

## Methods

### Chemicals

DL-*threo*-β-methylaspartic acid, β-glutamic acid, DL-3-aminobutanoic acid, L-lysine and L-2-aminoadipic acid (2AAA) were obtained from Sigma-Aldrich; L-β-lysine from Enamine (Riga, Latvia) and (*E*)-6-aminohex-2-enoic acid (6-AHEA) from Toronto Research Chemicals (North York, Canada).

### MAL selection, expression and purification

The gene sequences encoding for CaMAL, CtMAL and ChMAL (NCBI accession numbers AB005294, AAB24070 and ABB16231, respectively) were retrieved from the National Center for Biotechnology Information database, and codon optimized for expression in *Escherichia coli* [41]. CaMAL, CtMAL and ChMAL were synthetized by GeneScript (Piscataway, NJ, USA) and cloned in the vector pET28b so that a histidine tag was introduced in the C-terminal region of the genes.

pET-28b plasmids containing the MAL genes were transformed into *E*. *coli* BL21 (DE3) for protein expression. The cells were grown in LB medium with 50 μg/ml kanamycin and autoinduction medium with lactose [42] at 30°C, and 180 rpm for 16 h. The recollected cells were solubilized in 50 mM Tris-HCl (pH 8) containing 300 mM NaCl and 10 mM imidazole and sonicated (Branson Digital Sonifier, model 250) using an amplitude of 30%, in 7 cycles of 30 s, and then centrifuged for 20 min at 13500 rpm. The expressed proteins were purified using a 1-ml HisTrap column (GE Healthcare, Uppsala, Sweden) and 50 mM phosphate pH 8, 300 mM NaCl, 20 mM imidazole buffer. A gradient up to 500 mM imidazole was used to elute the purified protein.

The purity of the MAL enzymes was determined with SDS-PAGE (S9 Fig) and their concentration was measured by the absorbance at 280 nm. The extinction coefficient (ε_280_) and molecular weights (M) used to calculate the protein concentration were retrieved from the web-based tool ProtParam (https://web.expasy.org/protparam/). They were: ε_280_ = 74720 M^-1^cm^-1^ and M = 90.9 kDa for CaMAL, ε_280_ = 57760 M^-1^cm^-1^ and M = 91.0 kDa for CtMAL, and ε_280_ = 69680 M^-1^cm^-1^ and M = 98.5 kDa for ChMAL. Purified enzymes were stored in 50 mM potassium phosphate buffer (pH 8) containing 2 mM MgCl_2_ and 0.1 mM KCl, at -80°C.

### Enzyme activity assays

#### Steady-state kinetics

The deamination of 3-methylaspartate and aspartate was measured by monitoring mesaconate (ε_240_ = 3850 M^-1^ cm^-1^) and fumarate formation (ε_240_ = 2530 M^-1^ cm^-1^), respectively, in reaction buffer (0.25 M Tris pH 9 containing 20 mM MgCl_2_ and 1 mM KCl) at 30°C, using a SPECTROstar Nano microplate reader (BMG Labtech, Ortenberg, Germany). The 3-methylaspartate stock solution was made by dissolving DL-*threo*-3-methylaspartic acid (a 1:1 mixture of the enantiomers (2S,3S)-3-methylaspartic acid and (2R,3R)-3-methylaspartic acid) in 0.25 M Tris pH 9. The (2R,3R) enantiomer is neither a substrate nor an inhibitor of MAL [14]. All enzymatic activities were measured as initial velocities from linear increments in the absorbance due to the formation of the reaction product. Mean of three values and standard errors were obtained for the apparent affinity constant (Michaelis constant, *K*_m_) and the enzyme turnover (catalytic constant, *k*_cat_) by nonlinear least-squares fitting of the experimental measurements to the Michaelis-Menten model.

#### Spectrophotometric assays

In order to develop a reliable method for measuring the activity, the absorption spectra of the substrates and the expected products were recorded in the 200–800 nm range and used to calculate the extinction coefficient of the expected reaction products following the Beer–Lambert law. Then, it was decided to follow the deamination of β-lysine by monitoring 6-AHEA formation at 230 nm (ε_230_ = 5240 M^-1^cm^-1^), and the deamination of β-glutamate was followed by observing the formation of glutaconate (ε_230_ = 4350 M^-1^cm^-1^). The reaction mixtures contained 10–20 μg/ml MAL and 30 mM substrate in reaction buffer.

#### NMR to determine MAL activity on β-lysine

Reaction mixtures containing 1 mg/ml MAL and 60 mM β-lysine in reaction buffer were incubated at 30°C for 4 days. ^1^H NMR spectra were recorded at 25°C on a Bruker Avance III HD NMR spectrometer, operated at 800 MHz for proton detection. Ten per cent D_2_O was added to the reaction mixtures for NMR measurements.

#### HPLC assay to determine MAL activity on β-glutamate

Reaction mixtures containing 0.5–4.0 mg/ml MAL, 60 mM β-glutamate in reaction buffer were incubated at 30°C. Aliquots were withdrawn at different times (4 h, 24 h, 7 d and 14 d), and the formation of glutaconate (the product of β-glutamate deamination) was monitored at 210 nm using an HPLC system with a UV-4075 detector (Jasco, Japan) equipped with a Rezex RFQ-Fast Acid H+ (8%) LC column (Phenomenex, Aschaffenburg, Germany), maintained at 80°C. The mobile phase consisted of 5 mM H_2_SO_4_ at a flow rate of 0.8 ml min^−1^.

#### Assay to determine MAL activity on 3-aminobutanoic acid

Up to 1 mg/ml MAL and 60 mM 3-aminobutanoic acid were incubated in reaction buffer with 75 mM α-ketoglutarate, 4 mM NADH and 1 unit of L-glutamic dehydrogenase from bovine liver (GDH, Merck, Germany). Any ammonia formed as a product of MAL activity reacts with α-ketoglutarate and NADH in a reaction catalysed by GDH to form L-glutamate and NAD^+^. The conversion of NADH to NAD^+^ was followed (ε_340_ = 6220 M^-1^cm^-1^). Positive controls with both ammonia and 3-methylaspartate as substrates, and negative control reactions lacking MAL or 3-aminobutanoic acid, were included.

### Inhibition assays

The inhibition of CaMAL by β-lysine, β-glutamic acid, lysine and 2AAA, was investigated by measuring the activity and determining *K*_m_ and *k*_cat_ values in the presence of different concentrations of the potential inhibitors. The reaction mixtures contained 2 μg/ml CaMAL and 1.25–15 mM (2S,3S)-3-methylaspartic acid in reaction buffer. In order to determine the type of inhibition, double reciprocal (Lineweaver-Burk) plots and fitting of the data by non-linear regression to different inhibition models using OriginPro software (Version 2020, OriginLab corportation, Northampton, MA, USA) were used. The competitive inhibition model used the equation y = *k*_cat_ x / *K*_m_ (1 + *I*_c_/ *K*_i_) + x, where *k*_cat_ is the enzyme turnover (catalytic constant), *K*_m_ is the Michaelis constant, *I*_c_ is the concentration of the inhibitor, and *K*_i_ is the inhibition constant. The non-competitive inhibition model used the equation y = *k*_cat_ x / (1 + *I*_c_/ *K*_i_) (*K*_m_ + x).

### *In silico* saturation mutagenesis and experimental site-directed mutagenesis

An in-house pipeline was used for *in silico* high-throughput saturation mutagenesis based on the FoldX energy function [29, 30], also applied in previously studies [43–45]. This method was used to estimate the impact of mutations on the thermodynamic stability of CaMAL by performing the calculations starting from the CaMAL monomer in the apo form.

The crystallographic structure of the CaMAL dimer in complex with 3-methylaspartate (PDB entry 1KKR) considering only one monomer (chain A) was used. Modeller9.16 was used to replace the selenomethionines with methionines and to add two missing amino acids at the C-terminal of the protein. During modelling, all the protein atoms except for those of the two residues immediately next to each selenomethionine in the amino acidic sequence were restrained. Two hundred different models were generated, and the one with the lowest RMSD calculated over all the methionine atoms using their orientation in the starting structure as reference was selected. Pymol was then used to remove 3-methylaspartate, giving the apo structure. The RepairPDB function of FoldX was applied to the apo structure. An in-house Python script was used to obtain the starting structures for the mutagenesis scan, introducing all the possible 19 mutations at each position, and then calculating their effects in terms of ΔΔG (i.e. ΔG between the mutants and wild form). The BuildModel function of FoldX was used to perform five independent runs that were averaged. The common prediction error of FoldX is evaluated around 0.8 kcal/mol [46]. Twice the prediction error (i.e. 1.6 kcal/mol) was used as the threshold to define destabilizing and neutral mutations [43,45].

To obtain the single-mutant MAL variants, each mutation was introduced by PCR using the expression vector pET-28b (Novagen) harbouring the protein-coding sequence of CaMAL as a template and both a direct and a reverse primer, designed complementary to opposite strands of the same DNA region containing the desired mutation (S5 Table). Mutagenic PCR reactions were carried out in an Eppendorf Mastercycler Pro S using 10 ng template DNA, 250 μM of each dNTP, 125 ng of the direct and reverse primers (S5 Table), 2.5 units of Phusion HF polymerase (Thermo Scientific) and the manufacture’s reaction buffer. The reaction conditions were as follows: i) a “hot start” at 95°C for 1 min; ii) 18 cycles at 95°C for 50 s, 58°C for 50 s, and 68°C for 10 min; and iii) a final cycle at 68°C for 10 min. The mutated sequences were confirmed by DNA sequencing (Eurofins Genomics, Ebersberg, Germany).

### Molecular docking

Docking simulations [47] were carried out to predict the poses of six studied ligands in the pocket of the B chain of CaMAL (PDB entry 1KKR), and to estimate the binding affinity for the predicted poses. LeDock [48] and AutoDock Vina [49] software were used in these calculations. A recent benchmark study showed that the sampling methods implemented in LeDock (based on a combination of simulated annealing and evolutionary optimization) perform better than other methods. It was also pointed out that the scoring function of AutoDock Vina was the most accurate among the methods tested [48]. Thus, we first docked the ligands using LeDock, generating a different number of poses for each ligand, and then selected two of them which were re-scored using AutoDock Vina.

The structures of all the ligands were downloaded from the ZINC database [50]. As the first step, a region around the Mg^2+^ ion was defined as the binding site (the centre of a rectangular box, with the dimensions 60x60x60 Å^3^, was positioned on Mg^2+^). This box size is sufficiently large to encompass the natural substrate position and a large part of the protein, including the 8-fold α/β TIM barrel. A cut-off of 0.5 Å was used in LeDock to eliminate the poses that are too similar.The best poses were selected according to two criteria: i) the distances from the catalytic residues to the ligands, and ii) the RMSD between the ligands and 3-methylaspartate. Regarding the first criterion, the distances between 3-methylaspartate and the key residues in the binding pocket of CaMAL were used as reference. Four distances were calculated: between the nitrogen atom of the ε-amino group of the K331 and the carbon 3 of the ligands (4.16 Å in the reference structure); between the oxygen atoms in the carboxyl group of the ligands (in position 4 of 3-methylaspartate) and the nitrogen atom of the amide group of Q329 (3.08 Å in the reference structure), the ε nitrogen atom in the side chain of H194 (2.75 Å in the reference structure) and the Mg^2+^ ion (2.2 Å in the reference structure), respectively. To take into account the symmetry of the carboxyl group, the distances were measured considering both the oxygen atoms in the carboxyl group, and in the ensuing analyses only the shorter distances were considered. A distance cut-off of 4 Å was used to reject poses outside the binding pocket, assuming that at least three out of the four distances measured for the poses inside the pocket were below the cut-off.

The second criterion was based on the RMSD value between the ligands and the position of 3-methylaspartate in the reference structure. Therefore, a subset of atoms common to all the ligands and the natural substrate was considered: i.e., the two oxygen atoms and the carbon atom of the carboxyl group oriented towards the Mg^2+^ ion and the 3 following carbon atoms in the backbone of the ligands (Fig 5). Some of the ligands have two carboxylic groups, and we did not discriminate between the poses that present one or the other carboxylic group oriented towards the Mg^2+^ ion. The pose for the lowest RMSD relative to the position of 3-methylaspartate was selected for each ligand. The first predicted pose, according to LeDock scoring, was then redocked, and the binding affinity was compared with that of the one selected in AutoDock Vina.

The standalone version of Arpeggio, for calculating interatomic interactions classified in 15 different categories based on atom type, distance and angle terms [51], was used for the analysis of the interaction of each ligand with MAL. Chimera software was used for visual inspection and representation of the different complexes [52].

## Supporting information

S1 Fig Absorption spectra of the tested substrates and the corresponding products from a deamination reaction. (a) Absorption spectra of β-lysine and its deamination product 6-AHEA. (b) Absorption spectra of β-glutamic acid and its deamination product glutaconic acid. Inserts show the absorption spectra in the region 220–280 nm. All the compounds had a concentration of 200 μM.
(TIF)

S2 Fig CaMAL, CtMAL and ChMAL activity towards β-lysine. The formation of 6-AHEA (as product of β-lysine deamination) was monitored at 230 nm at different times (30 min, dark grey bars; 3 h, black bars; 24 h, light grey bars; and 7 d, white bars). Two negative controls were made: i) a control with MAL incubated in reaction buffer without substrate (CaMAL, CtMAL or ChMAL control) and ii), a control in which the substrate was incubated in the reaction buffer without enzyme (Control Substrate). A Positive Control was included in which the MAL activity towards 3-methylaspartic acid was followed monitoring mesaconate formation at 230 nm.
(TIF)

S3 Fig CaMAL single mutant variants activity towards β-lysine. a, CaMAL C361A; b, CaMAL C361S; c, T360A; d, T360S; e, L384A. The formation of 6-AHEA was monitored 230 nm at different times (10 min, black bars; 40 min, white bars; 6 h, grey bars; 24 h, light grey bars, 48 h, dark grey bars, and 7 d, white dotted bars). Two negative controls were made, CE in which β-Lysine (β-Lys) was incubated in the reaction buffer without enzyme; and CS in which only the enzyme was incubated in the reaction buffer. One positive control was made (C+) in which the variants activity towards 3-methylaspartic acid was followed by monitoring mesaconic acid formation at 230 nm.
(TIF)

S4 Fig HPLC activity assay for detecting MAL activity on β-glutamic acid. a, chromatograms obtained for samples containing β-glutamic acid (BG) and the deamination product glutaconate (Glut) in reaction buffer. c, e and g show the chromatograms for the reactions with CaMAL, CtMAL and ChMAL, respectively with 60mM of β-glutamic acid in reaction buffer. d, f and h show the chromatograms of the negative controls (without substrate) with CaMAL, CtMAL and ChMAL in reaction buffer. b shows the chromatogram corresponding to β-glutamic acid incubated in reaction buffer (and no enzyme). In b, the peak corresponding to β-glutamic acid was slightly displaced to the right and increased over time. This phenomenon was also observed in the MAL reactions (panels c, e and g).
(TIF)

S5 Fig Inhibition assay with lysine. Double reciprocal (Lineweaver-Burk) plot of the initial velocity versus 3-methylaspartate concentration with increasing lysine concentration. Results are the means of three replicates with 95% confidence limits. Lysine chemical structure is shown.
(EPS)

S6 Fig Box plot of the RMSD values. Values with median and outliners, of selected atoms that the ligands share with the natural substrate (relative to the crystal structure position of natural substrate). In the legends, we included the number of poses obtained for each ligand using *LeDock*.
(EPS)

S7 Fig The catalytic pocket of CaMAL (shown as light brown cartoon) with the natural substrate (upper part, PDB entry 1KKR, shown as pink sticks) and one example of the complexes with 2-aminoadipic acid (light blue sticks), 3-aminobutanoic acid (blue sticks), aspartic acid (light orange sticks) and lysine (pink sticks) obtained by docking calculations. We illustrate the different orientation of the ligands with respect to the hydrophobic residues in the catalytic pocket of CaMAL (residues L384, F170, Y356 shown as orange sticks) and the residue Q73, Q172,H194, Q329, T360, (shown as blue sticks) and K331 (shown as brown sticks) which take part in the hydrogen bond network.
(EPS)

S8 Fig Arpeggio results. In the left part of the figure, we show the summary of the Arpeggio interactions calculated for the natural substrate and for the other substrates investigated. We included in the analysis both the first pose (as predicted by LeDock) and the selected pose (the one with the lowest RMSD value compared to the positioning of the natural substrate in the reference structure). In the case that the substrate has two carboxylic groups, we show results for both orientations. In the right part, we show a graphical representation of the interactions calculated for natural substrate, indicating the residues of MAL which are included in those interactions.
(EPS)

S9 Fig SDS-PAGE of the purified CaMAL (45 kDa), CtMAL (45 kDa) and ChMAL (49 kDa). The protein marker can be found in lane 1.
(TIF)

S1 Table *In silico* saturation mutagenesis scan results used to design five single mutant variants of MAL. Mutations contained by the single variants and ΔΔG values obtained for different mutations around the MAL catalytic pocket in the presence of lysine (binding ΔΔGs) and in unbound state of the protein (stability ΔΔGs).
(TIF)

S2 Table Kinetic constants for deamination of 3-methylaspartate by CaMAL and the five designed single mutant variants. Reactions were carried out at 30°C in 0.5 M Tris (pH 9), 20 mM MgCl_2_, 1 mM KCl. Means and 95% confidence limits are shown.
(TIF)

S3 Table Measurement of ammonia formed in the reactions of MAL with 3-aminobutanoic acid after different times of incubation. 1mg/ml of MAL and 60 mM of 3-aminobutanoic acid was incubated in reaction buffer (250 mM Tris pH 9, 20 mM MgCl_2_, 1 mM KCl) with 75 mM α-ketoglutarate, 4 mM of NADH and 1 unit of GDH. The conversion of NADH to NAD^+^ was followed spectrophotometrically at 340 nm (ε_340_ = 6220 M^-1^cm^-1^). The ammonia quantified is expressed as μg/ml. The control samples contained 3-aminobutanoic acid 60 mM in reaction buffer. Means and 95% confidence limits.
(TIF)

S4 Table Binding affinities. The binding affinities calculated in the AutoDockVina program for two different docking poses: the first predicted pose, according to LeDock scoring, and the selected pose i.e. the pose with the lowest RMSD value relative to the positioning of the natural substrate in the reference structure (PDB entry 1KKR).
(TIF)

S5 Table Primers used for the production of the single MAL mutant variants. Only the direct sequences with indication of the changed triplets (underlined) and the mutations introduced (bold) are listed.
(TIF)
