# Supplementary figures and images for: Structure-function investigation of 3-methylaspartate ammonia lyase reveals substrate molecular determinants for the deamination reaction
Source: PLoS One. 2020 May 21;15(5):e0233467. doi: 10.1371/journal.pone.0233467 (PMC7241714; doi:10.1371/journal.pone.0233467)

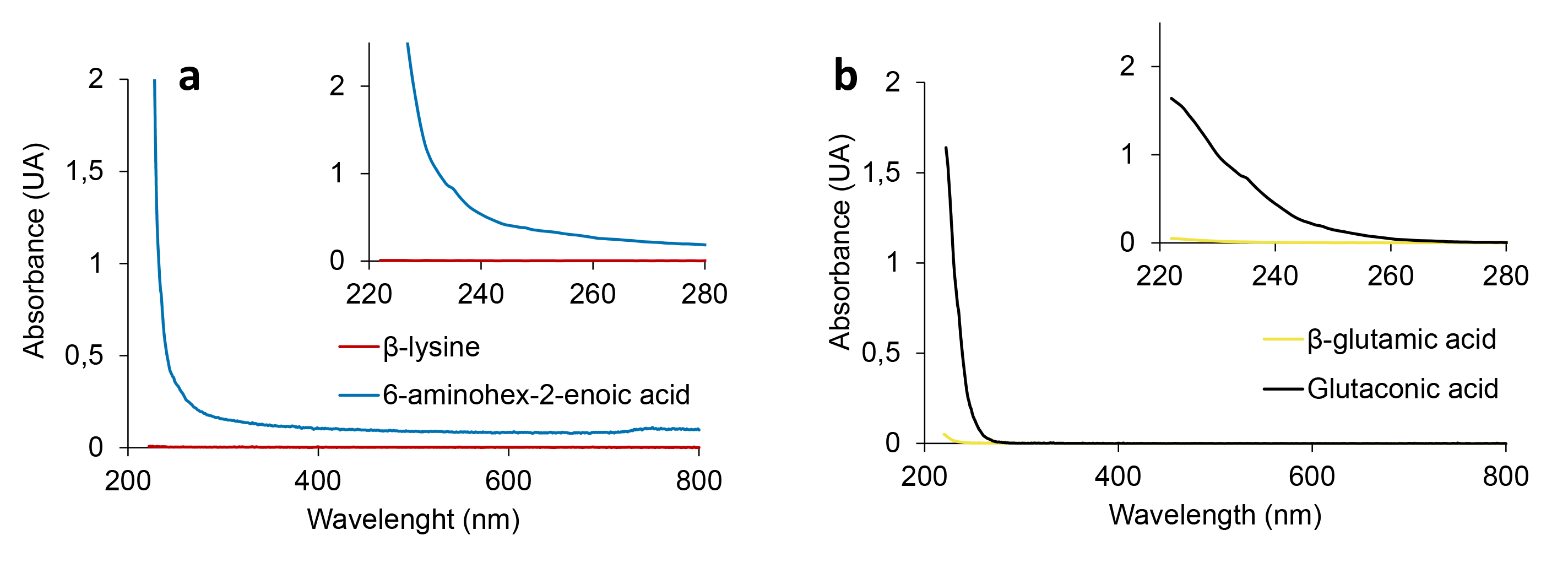

Supplement: S1 Fig — (a) Absorption spectra of β-lysine and its deamination product 6-AHEA. (b) Absorption spectra of β-glutamic acid and its deamination product glutaconic acid. Inserts show the absorption spectra in the region 220–280 nm. All the compounds had a concentration of 200 μM. (TIF) [file pone.0233467.s001.tif]

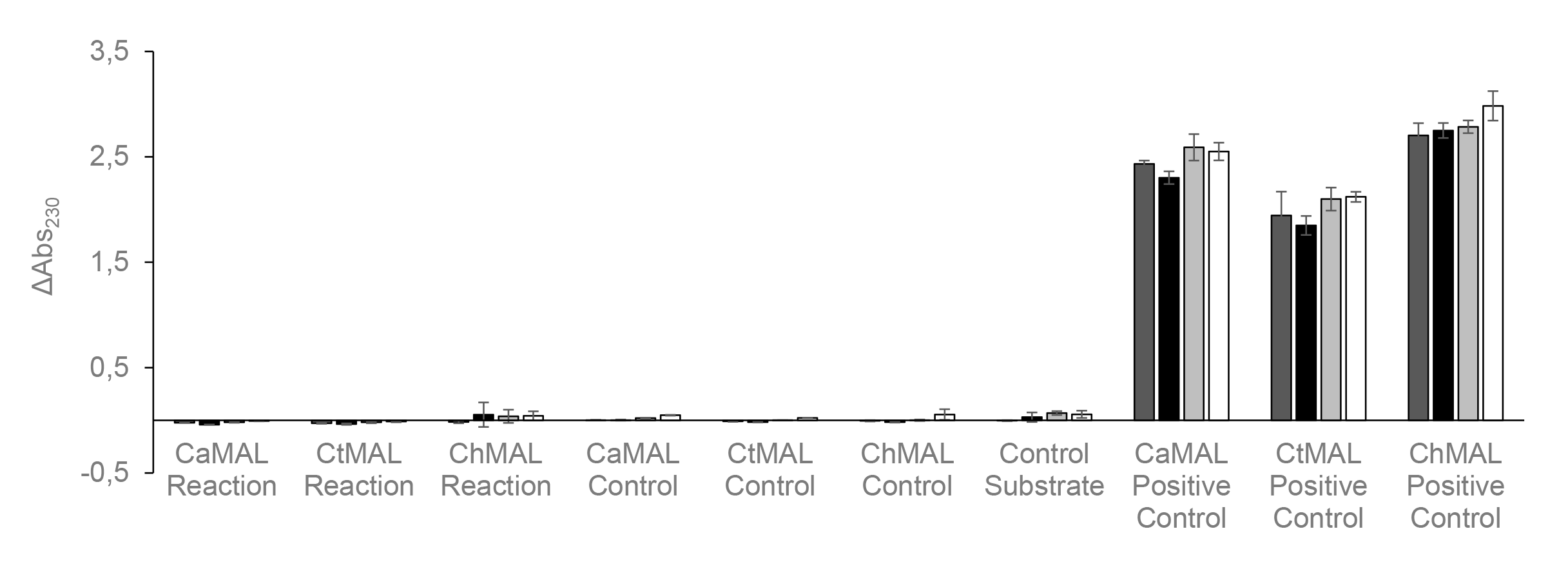

Supplement: S2 Fig — The formation of 6-AHEA (as product of β-lysine deamination) was monitored at 230 nm at different times (30 min, dark grey bars; 3 h, black bars; 24 h, light grey bars; and 7 d, white bars). Two negative controls were made: i) a control with MAL incubated in reaction buffer without substrate (CaMAL, CtMAL or ChMAL control) and ii), a control in which the substrate was incubated in the reaction buffer without enzyme (Control Substrate). A Positive Control was included in which the MAL activity towards 3-methylaspartic acid was followed monitoring mesaconate formation at 230 nm. (TIF) [file pone.0233467.s002.tif]

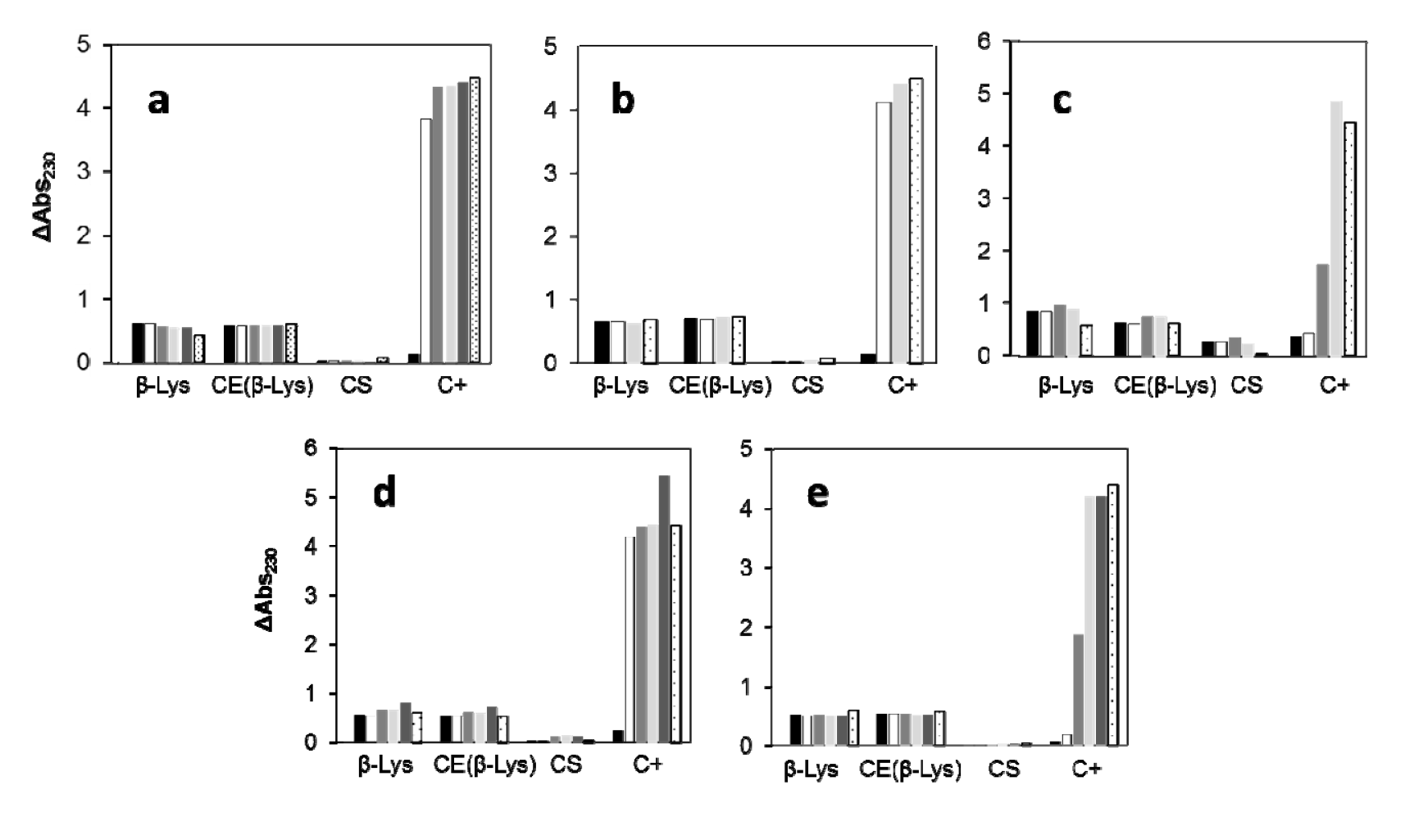

Supplement: S3 Fig — a, CaMAL C361A; b, CaMAL C361S; c, T360A; d, T360S; e, L384A. The formation of 6-AHEA was monitored 230 nm at different times (10 min, black bars; 40 min, white bars; 6 h, grey bars; 24 h, light grey bars, 48 h, dark grey bars, and 7 d, white dotted bars). Two negative controls were made, CE in which β-Lysine (β-Lys) was incubated in the reaction buffer without enzyme; and CS in which only the enzyme was incubated in the reaction buffer. One positive control was made (C+) in which the variants activity towards 3-methylaspartic acid was followed by monitoring mesaconic acid formation at 230 nm. (TIF) [file pone.0233467.s003.tif]

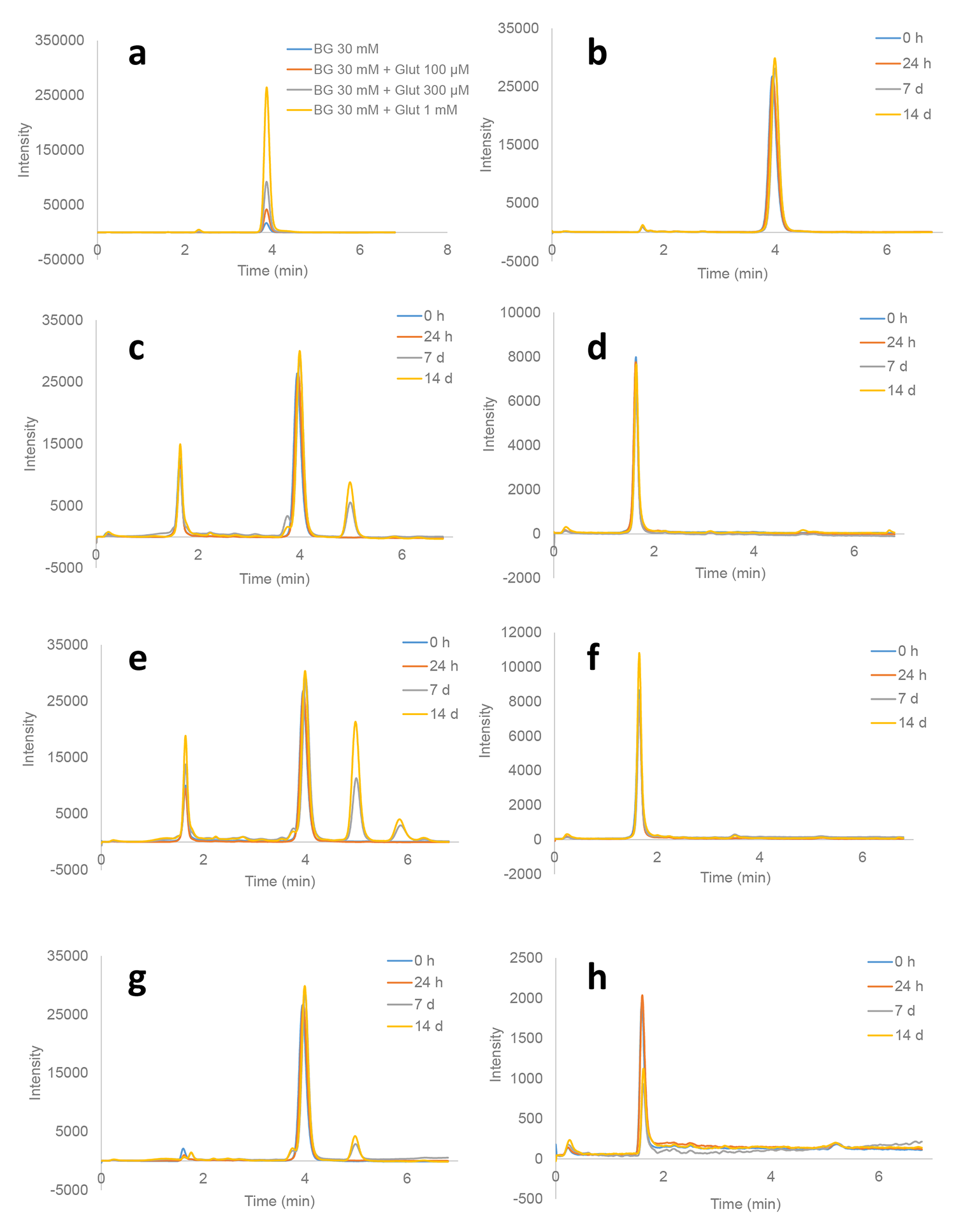

Supplement: S4 Fig — a, chromatograms obtained for samples containing β-glutamic acid (BG) and the deamination product glutaconate (Glut) in reaction buffer. c, e and g show the chromatograms for the reactions with CaMAL, CtMAL and ChMAL, respectively with 60mM of β-glutamic acid in reaction buffer. d, f and h show the chromatograms of the negative controls (without substrate) with CaMAL, CtMAL and ChMAL in reaction buffer. b shows the chromatogram corresponding to β-glutamic acid incubated in reaction buffer (and no enzyme). In b, the peak corresponding to β-glutamic acid was slightly displaced to the right and increased over time. This phenomenon was also observed in the MAL reactions (panels c, e and g). (TIF) [file pone.0233467.s004.tif]

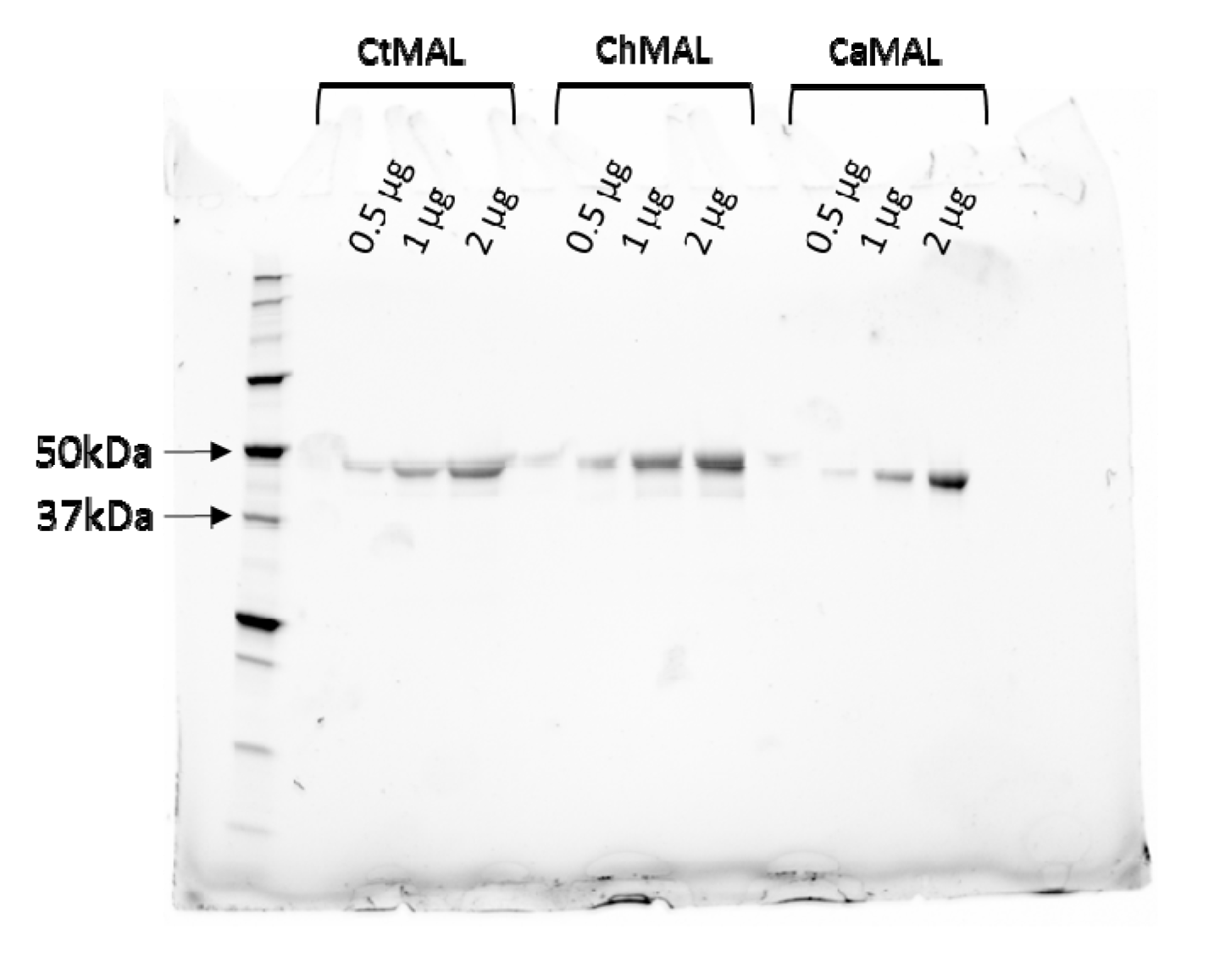

Supplement: S9 Fig — The protein marker can be found in lane 1. (TIF) [file pone.0233467.s009.tif]

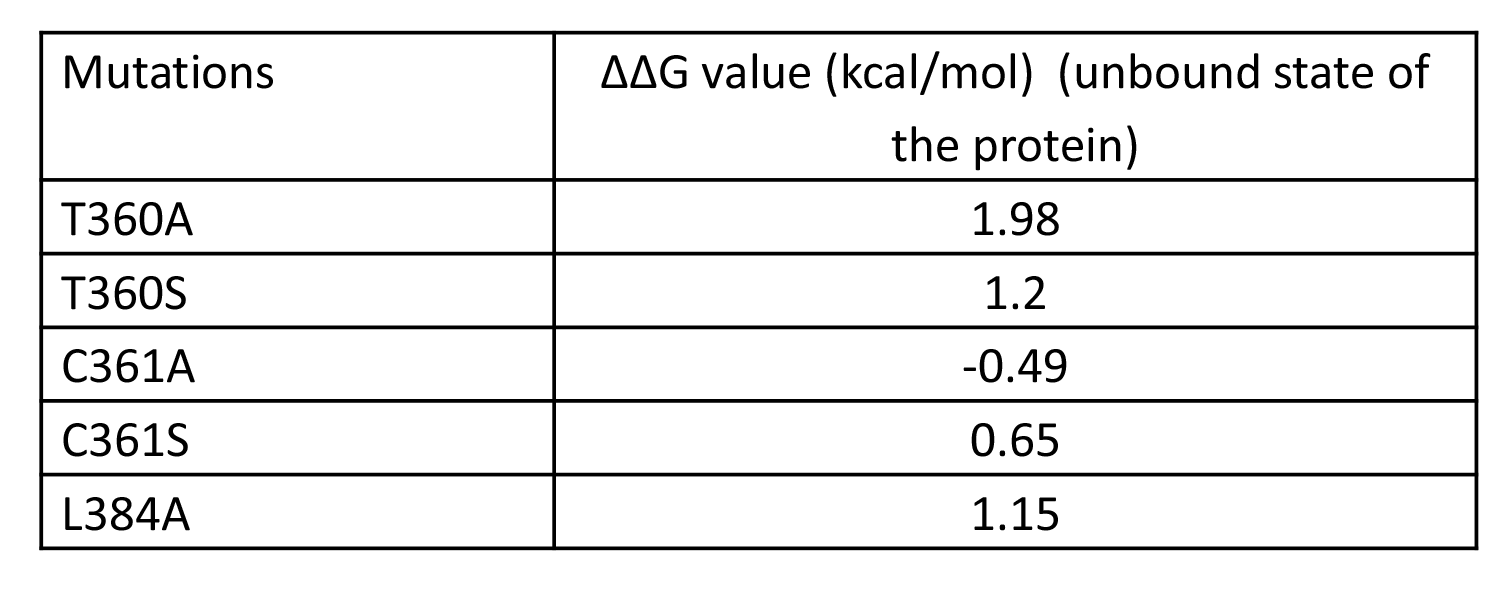

Supplement: S1 Table — Mutations contained by the single variants and ΔΔG values obtained for different mutations around the MAL catalytic pocket in the presence of lysine (binding ΔΔGs) and in unbound state of the protein (stability ΔΔGs). (TIF) [file pone.0233467.s010.tif]

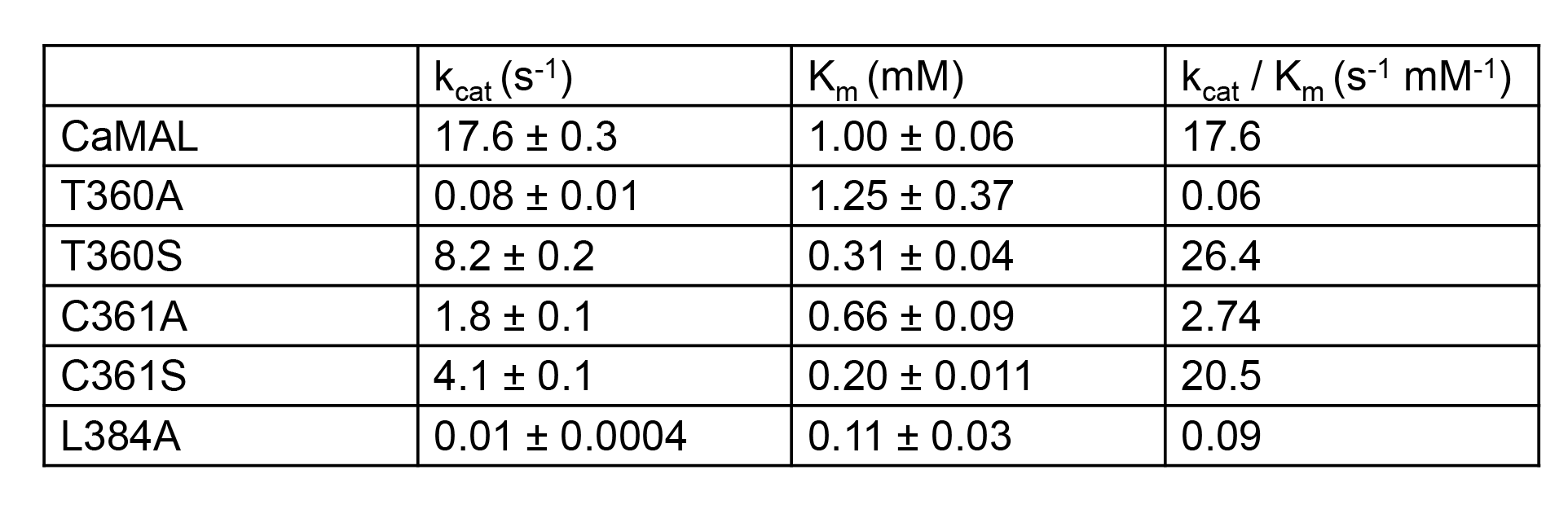

Supplement: S2 Table — Reactions were carried out at 30°C in 0.5 M Tris (pH 9), 20 mM MgCl2, 1 mM KCl. Means and 95% confidence limits are shown. (TIF) [file pone.0233467.s011.tif]

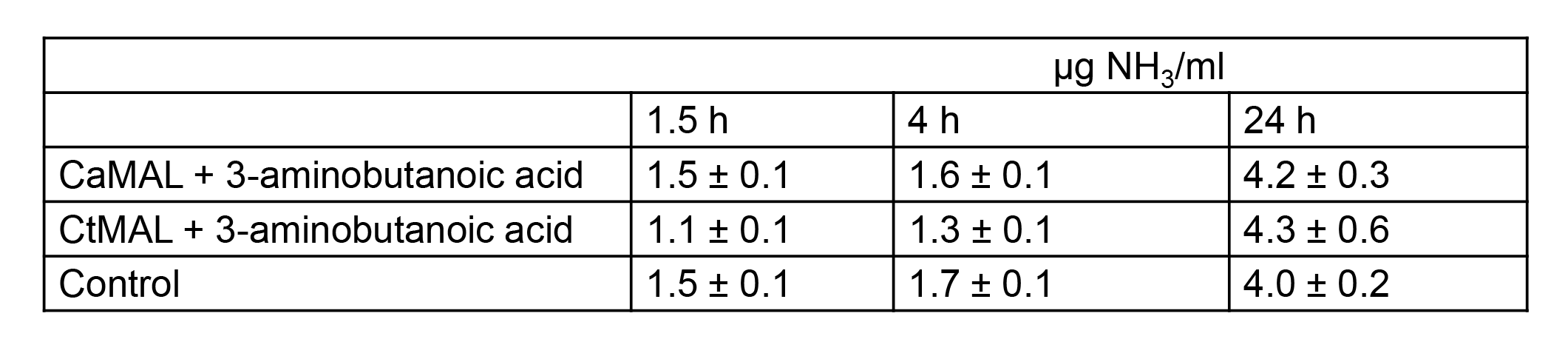

Supplement: S3 Table — 1mg/ml of MAL and 60 mM of 3-aminobutanoic acid was incubated in reaction buffer (250 mM Tris pH 9, 20 mM MgCl2, 1 mM KCl) with 75 mM α-ketoglutarate, 4 mM of NADH and 1 unit of GDH. The conversion of NADH to NAD+ was followed spectrophotometrically at 340 nm (ε340 = 6220 M-1cm-1). The ammonia quantified is expressed as μg/ml. The control samples contained 3-aminobutanoic acid 60 mM in reaction buffer. Means and 95% confidence limits. (TIF) [file pone.0233467.s012.tif]

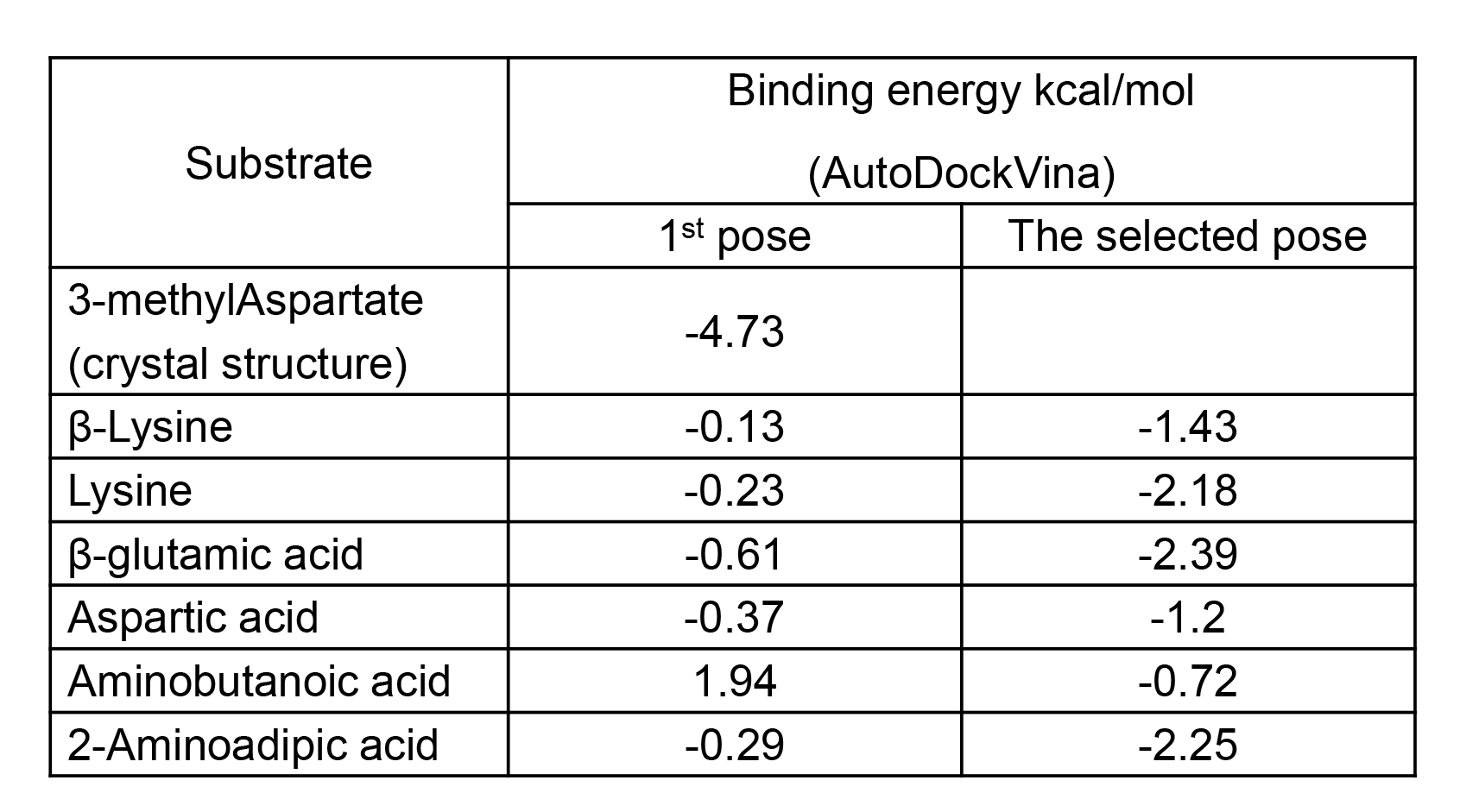

Supplement: S4 Table — The binding affinities calculated in the AutoDockVina program for two different docking poses: the first predicted pose, according to LeDock scoring, and the selected pose i.e. the pose with the lowest RMSD value relative to the positioning of the natural substrate in the reference structure (PDB entry 1KKR). (TIF) [file pone.0233467.s013.tif]

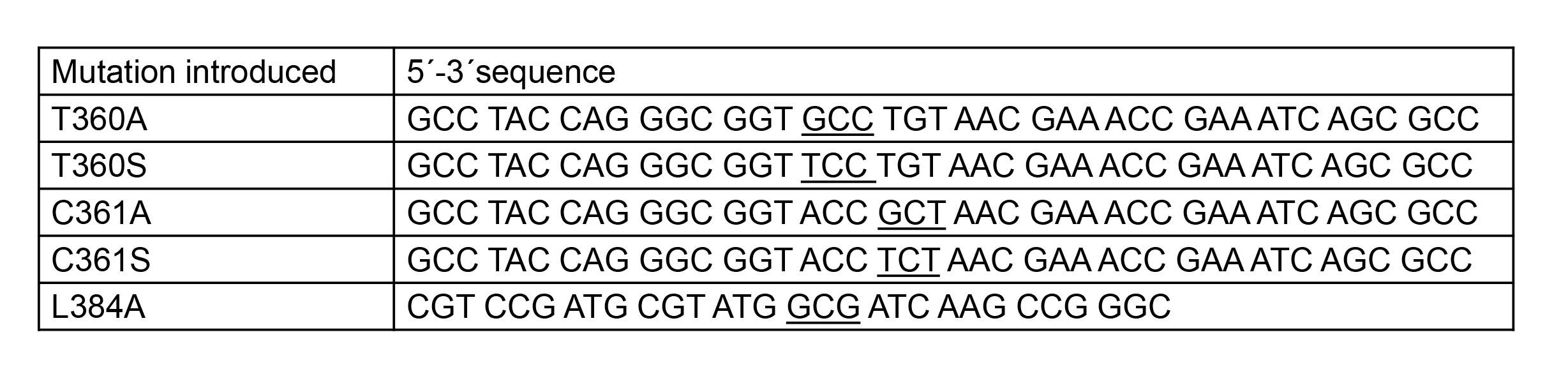

Supplement: S5 Table — Only the direct sequences with indication of the changed triplets (underlined) and the mutations introduced (bold) are listed. (TIF) [file pone.0233467.s014.tif]
